# Prebiotics, faecal transplants and microbial network units to stimulate biodiversity of the human gut microbiome

**DOI:** 10.1111/1751-7915.12049

**Published:** 2013-04-18

**Authors:** Pieter Van den Abbeele, Willy Verstraete, Sahar El Aidy, Annelies Geirnaert, Tom Van de Wiele

**Affiliations:** 1 Laboratory of Microbial Ecology and Technology (LabMET), Ghent UniversityCoupure Links 653, 9000, Ghent, Belgium; 2 Department of Experimental Oncology, European Institute of OncologyVia Ripamonti 435, 20141, Milan, Italy

## Abstract

Accumulating evidence demonstrates the intimate association between human hosts and the gut microbiome. Starting at birth, the sterile gut of the newborn acquires a diverse spectrum of microbes, needed for immunological priming. However, current practices (caesarean sections, use of formula milk) deprive newborns from being exposed to this broad spectrum of microbes. Unnecessary use of antibiotics and excessive hygienic precautions (e.g. natural versus chlorinated drinking water) together with the Western diet further contribute to a decreased microbial diversity in the adult gut. This has been correlated with recurrent *Clostridium difficile* infection, inflammatory bowel diseases and obesity, among others. A healthy gut microbiome is thus characterized by a diverse network of metabolically interacting microbial members. In this context, we review several existing and novel approaches to manage the gut microbiome. First, prebiotic compounds should be re-defined in the sense that they should enhance the ecological biodiversity rather than stimulating single species. Recent studies highlight that structurally different polysaccharides require specific primary degraders but also enhance a similar network of secondary degraders that benefit from cross-feeding. A faecal transplantation is a second approach to restore biodiversity when the microbiota is severely dysbiosed, with promising results regarding *C. difficile*-associated disease and obesity-related metabolic syndromes. A final strategy is the introduction of key microbial network units, i.e. pre-organized microbial associations, which strengthen the overall microbial network of the gut microbiome that supports human health.

## Introduction

**Figure 1:**
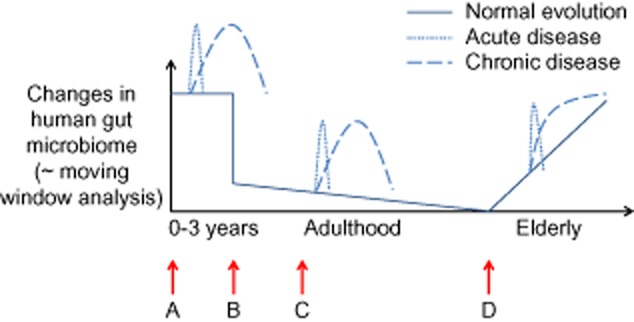
Changes in the gut microbiome over the course of a human lifespan. After drastic changes in early childhood, the gut microbiota stabilizes to an adult microbial composition. During the elderly and acute or chronic diseases, specific states of dysbiosis have been documented. Future ecosystem approaches to restore intestinal imbalances may thus target four distinct populations (A: newborns; B: young adults; C: diseased subjects; D: elderly).

Over the last decades, specific mechanisms of how the human gut microbiome determines human health or disease states have been elucidated (Round and Mazmanian, 2009). It has become clear that the dynamic interaction with these intestinal microbes can be divided in different stages over the human lifespan (Fig. 1). Drastic changes occur during the first years of life as the newborn gut progresses from a sterile environment to a densely populated microbial habitat. This gut ecosystem exerts restrictive selection on its microbial inhabitants as only microbes that are capable of establishing a mutualistic relation with the host are maintained (Backhed *et al*., 2005; Ley *et al*., 2008). In human infancy and early childhood, the complex microbial ecosystem is formed through the successive establishment of different bacterial groups; aerotolerant bacteria establish first, followed by more and more strict anaerobes, as observed in ex-germfree reductionist animal models (Adlerberth and Wold, 2009). As a result, the infant gastrointestinal microbiota is quite variable in its composition and relatively unstable over time. On average, 3 years after birth the microbial community consists of a mixture of microbes that is largely similar to that found in the adult intestine (Yatsunenko *et al*., 2012). At that time, the complex microbiota is predominantly colonized by obligate anaerobes to provide a strong barrier against the establishment and proliferation of new bacterial groups, in a phenomena known as colonization resistance (Vollaard and Clasener, 1994). The colonizing microbes can rapidly shape themselves in response to changes in host environment. Likewise, when the host environment changes, the immune system and metabolic profile must adjust to these fluctuations in order to keep a mutualistic relationship with its microbiota. Although individual-specific and relatively stable in younger healthy adults, the microbiota in elderly displays greater inter-individual variation (Claesson *et al*., 2011). Such high variation correlates with immunosenescence, which characterizes the ageing process (Claesson *et al*., 2012). Moreover, once its symbiotic coexistence is disrupted during acute (e.g. invasion of pathogens or antibiotic treatment) or chronic conditions (e.g. inflammatory bowel diseases and obesity), the microbial community may become a major threat to the host.

This raises the question of how to restore the microbial dysbiosis and the resulting imbalance in the host–microbe symbiotic relationship when things go wrong. Existing approaches to manage the human gut microbiome include functional foods such as probiotics, prebiotics and synbiotics. While probiotics are beneficial live microorganisms (FAO/WHO, 2002), prebiotics are indigestible food compounds that selectively stimulate specific beneficial microbial genus(era)/species already present in the gut (Gibson and Roberfroid, 1995; Roberfroid *et al*., 2010). Unfortunately, antibiotics are still commonly prescribed causing severe disturbances in the microbiome. A very promising therapeutic approach is the use of faecal transplants, which allows to install a healthy, diverse gut microbiome in a human subject that suffered from a severely dysbiosed gut microbiota. In this mini-review, we emphasize the importance of biodiversity and, in this context, re-define the current prebiotic concept. We also propose a novel ecosystem-based approaches to restore host–microbial imbalances in disease based on the success of faecal transplants, i.e. by providing specific microbial network units.

## The biodiversity concept

Biodiversity is generally considered crucial for the well-functioning of an ecosystem. This is also valid for the microbial ecosystem of the human gut where a decreased diversity has been correlated with disease states such as recurrent *Clostridium difficile* infection (Chang *et al*., 2008), Crohn's disease (Manichanh *et al*., 2006) and obesity (Turnbaugh *et al*., 2009). A recent study by Yatsunenko and colleagues (2012) elucidated that US residents have a markedly less diverse microbiota than human subjects living in ancient geographic locations.

This lower microbial diversity is caused by several typical Western society practices. Everything starts at birth, as caesarean sections are becoming a common mode of delivery. In 2010, almost one-third of the births in the USA was through caesarean delivery (Martin *et al*., 2012). This deprives the newborn from being exposed to a broad spectrum of vaginal microbes resulting in lower intestinal microbial diversity in the gut microbiome of the newborn (Biasucci *et al*., 2012). Moreover, breastfeeding is often replaced by formula milk despite the fact that breast milk is essential for the early microbial development by providing nutrients for early-life symbionts (e.g. bifidobacteria) (Sela and Mills, 2010) and even by providing live bacteria (Cabrera-Rubio *et al*., 2012). Misuse of antibiotic treatments (Dethlefsen *et al*., 2008) further contributes to the decreased microbial diversity in the human gut microbiome. Another microbial exposure route that has been decreasing in the last decades is exposure via the drinking water. In most countries, drinking water is sterilized by the addition of chlorine. In countries such as the Netherlands and Switzerland water is not disinfected. Although drinking water may not contain real gut commensals, drinking chlorinated water instead of non-disinfected water raises questions regarding immune system education capacity. The question is often raised whether public health, fitness or capacity to excel might relate to the issue of natural versus chlorinated water, but is still not documented properly. This issue certainly deserves appropriate study.

**Figure 2:**
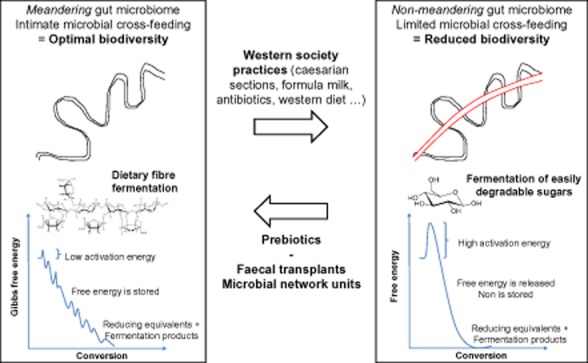
The stepwise release of carbohydrate-derived energy by gut microorganisms can be compared by a meandering river in which the water potential energy is gradually lowered. Such an environment creates many functional niches thereby selecting for a high microbial diversity, similar to a high biological diversity in meandering river ecosystem. Shortcuts through microbial metabolic meanders in the human gut can be compared with the canalization of a meandering river. Such environment entails a limited amount of functional niches thereby selecting for a limited microbial diversity.

The Western diet that is unbalanced and too homogeneous in composition also contributes to a lower microbial diversity and eventually does not support the co-evolved host–microbe partnership (De Filippo *et al*., 2010). The non-digestible fraction consists of substrates that do not require complex metabolic interactions between members of the gut microbial ecosystem. This results in rapid release of nutritional energy by a limited amount of microbes. In contrast, diets that have a high dietary fibre content do support microbial diversity as this stimulates microbial interactions through which nutrient-derived energy is harvested in different stages by different microbial members. This can be compared with the dissipation of potential energy over several meanders in a river system. We therefore state that a healthy well-structured microbiome is characterized by a meandering metabolism (Fig. 2). Such slow release of nutritional energy is characteristic for high fibre diets which may protect against inflammation and non-infectious colonic diseases (De Filippo *et al*., 2010). In contrast, the typical Western diet results in dissipation of the nutrient-derived energy through shortcuts in such metabolic meanders. In terms of the ecological r/K-selection theory (Pianka, 1970), this selects for a higher abundance of r-strategists: microorganisms that have a low substrate affinity but that display high growth rates when easily degradable substrates are abundantly available. This also results in a depletion of K-strategists: microorganisms that have a high substrate affinity but that display low growth rates as they rely on other microbial members that participate in the metabolic ‘meandering’ network.

Starting at birth, the human body is exposed to a lower diversity of microbial antigens as a consequence of the reported typical Western society practices. This results in a weakened immune regulation that depletes the gut mucosal barrier and predisposes the human host to Western diseases that have been increasing since the beginning of the 20th century.

## Towards ecosystem approaches to restore the diversity of the human microbiome: prebiotics, faecal transplants and microbial network units

Given the importance of biodiversity in the gut microbiome for human health, therapeutic strategies that aim at improving health should incorporate this biodiversity concept. Besides redefining existing concepts such as the prebiotic concept, this may lead to novel ecosystem-based approaches such as supplementation of essential microbial network units.

First, the concept of prebiotic compounds may need to be revised. Prebiotics have been defined as ‘indigestible food compounds that selectively stimulate the growth and/or activity of one or a limited number of microbial genus(era)/species in the gut that confer(s) health benefits to the host’ (Gibson and Roberfroid, 1995; Roberfroid *et al*., 2010). While stimulation of bifidobacteria or lactobacilli is generally regarded as beneficial, this concept seems too narrow. A new, more accurate definition for a prebiotic compound may be ‘an indigestible food compound that stimulates the ecological biodiversity in the human gut microbiome’. Indeed, a recent study using a novel molecular tool with high-phylogenetic resolution indicated that prebiotic compounds such as inulin (IN) and long-chain arabinoxylans (LC-AX) both increase the abundance of specific primary degraders. Despite the very different structure of IN and LC-AX, both compounds also increased a similar spectrum of secondary degraders that benefit through cross-feeding with the primary degrader (Table 1) (Van den Abbeele *et al*., 2011). Such microbes that increased in both treatments mostly belonged to relatives from the *Clostridium* clusters IV and XIVa. Although primary degraders are essential to initiate the breakdown of the specific polysaccharides, the stimulation of these microbial networks/meanders increases the microbial diversity and may be more beneficial than the increase of a specific primary degrader.

**Table 1:** Complex polysaccharides that arrive in the colon [such as long-chain arabinoxylans (LC-AX) or inulin (IN)] increase the abundance of specific primary degraders but importantly, they also increase a similar cohort of secondary degraders that benefit through cross-feeding. This increases the microbial diversity of the human microbiome.

		Percentual abundance (%)		
Higher taxonomic group	Bacterial group	Control	LC-AX	IN
*Clostridium* cluster IV	*Subdoligranulum variable*-like	0.50 ± 0.06^a^	1.44 ± 0.37^b^	1.59 ± 0.29^b^
*Clostridium* cluster XIVa	*Clostridium colinum*-like	0.62 ± 0.04^a^	3.20 ± 0.10^b^	3.65 ± 0.37^b^
	*Clostridium sphenoides*-like	1.63 ± 0.27^a^	3.36 ± 0.17^b^	3.23 ± 0.21^b^
	*Eubacterium rectale*-like	0.96 ± 0.16^a^	4.72 ± 0.36^b^	5.95 ± 0.69^b^
	*Lachnospira pectinoschiza*-like	0.98 ± 0.11^a^	2.54 ± 0.18^b^	3.16 ± 0.19^c^
	*Lachnobacillus bovis*-like	1.06 ± 0.21^a^	2.72 ± 0.60^ab^	3.11 ± 0.47^b^
	*Roseburia intestinalis*-like	0.54 ± 0.05^a^	4.43 ± 1.10^b^	5.90 ± 1.11^b^

This table contains an example of such a cohort of secondary degraders, as identified during a study with humanized rats, treated with LC-AX or IN Van den Abbeele *et al*., 2011). The abundance (%) of bacterial groups (belonging to higher taxonomic groups) are based on the HITChip analysis. Values indicated with a different superscript are significantly different (a, b or c; *n* = 4).

A second approach to restore microbial diversity when a human microbiota is severely disturbed is a faecal transplantation (Shahinas *et al*., 2012). This approach has already been applied to treat *C. difficile*-associated disease (Khoruts *et al*., 2010; Guo *et al*., 2012), which is thought to result from persistent disruption of the commensal gut microbiota and which is considered as an important cause of antibiotic-induced diarrhoea and colitis. Moreover, faecal transplantation from lean individuals to individuals suffering from metabolic syndrome, displayed increased insulin sensitivity corresponding with stimulation of microbial groups that are known to benefit from cross-feeding (metabolic meandering) such as butyrate-producing microbes of the *Clostridium* clusters IV and XIVa (Vrieze *et al*., 2012). Faecal transplants thus seem to be promising techniques to restore microbial diversity in microbial communities that are severely dysbiosed. However, given the high microbial complexity of human faeces, faecal samples require thorough risk assessment before they can be applied as a faecal transplant. Therefore, faecal transplants are only applied in severe cases while otherwise antibiotics are still a common practice. To guarantee a more stable and diverse microbial composition during such antibiotic treatments, the current recommendation is to consume yoghourt or cheese (often containing a single microbial species) during these treatments. The current state of the art should allow to go beyond these recommendations and design much better products.

Therefore, based on the concept of faecal transplants, we introduce a third concept to remediate a dysbiosed microbiota or to assist microbiome development during different stages of a human lifespan: intestinal microbial network units. Such a network unit contains a consortium of microbes that is capable of performing a specific function, as we will demonstrate according to three different examples: degradation of specific complex polysaccharides (e.g. starch, LC-AX or mucins), induction of specific immune pathways (e.g. Treg cells) or a mixture of essential symbionts for a specific human life stage (e.g. microbial mix for newborns).

The first example considers the fermentation of otherwise indigestible polysaccharides to health-promoting short-chain fatty acids. This microbial metabolic function is so beneficial that it is assumed to be the main evolutionary driving force to include microorganisms in the gastrointestinal tract and to acquire carefully designed defence mechanisms (McFall-Ngai, 2007). The diverse spectrum of glycans, which is present in the human colon, is derived from the diet (resistant starch, LC-AX, IN) or the host (mucins). To construct a microbial network unit for these specific glycans, one needs to consider the concept of a king and his court. First, there is the specific primary degrader or keystone species for this glycan (‘king’). For many of the diverse spectrum of glycans that are present in the colon, such keystone species have been proposed. As an example, resistant starch requires the presence of *Ruminococcus bromii* (Ze *et al*., 2012), LC-AX are specifically degraded by *Bifidobacterium longum* (Van den Abbeele *et al*., 2011) and *Akkermansia muciniphila* seems a crucial species for the initial breakdown of mucins (Belzer and de Vos, 2012). To produce an industrial product, i.e. an effective microbial network unit, one also needs to provide the primary degrader (‘king’) with its cross-feeding microbes (‘court’). This may be achieved using conventional *in vitro* fermentors which can be inoculated with relevant keystone species and a mixture of cross-feeding microbes (e.g. relatives of the microbial groups listed in Table 1). After inoculation and stabilization, such a microbial network unit for specific functions can be attained and produced at large scale.

As a second example, microbial network units may also be constructed for specific immunomodulatory functions such as induction of the regulatory arm of colonic T cells. A recent study by El Aidy and colleagues (2012a) reported on the dynamic changes in the regulation of intestinal homeostasis during microbial colonization. During the process of microbial colonization in germfree mice, inflammatory tissue responses were avoided by the induction of the regulatory arm of T cells, including (among others) Foxp3 and Il10 markers for tolerance-promoting Tregs that were induced, especially during the later stage of colonization of the colon. The expression of these tolerance markers paralleled the colonization of several *Clostridium* cluster IV and XIVa species (El Aidy *et al*., 2012b). The latter bacterial groups were previously reported to stimulate the expression of Tregs (Atarashi *et al*., 2011), potentially related to their strong capacity to colonize the mucin-associated microbiota (Van den Abbeele *et al*., 2012). Moreover, these *Clostridium* cluster IV and XIVa species represent only a small proportion of the microbial community in the inflammatory bowel disease patients (Frank *et al*., 2007). Constructing a microbial network unit that contains these species may thus allow reaching a microbial network unit that specifically induces colonic Tregs that may be relevant for administration to inflammatory bowel disease patients.

Third, microbial network units may also be relevant with respect to the different stages of a human lifespan (Fig. 1). As discussed above, current Western society practices such as caesarean sections and the use of formula milk, contribute to early host–microbial imbalances. When designing a microbial mix for babies, it will be important that besides the presence of well-known early-life symbionts such as *Bifidobacterium* sp., also bacteria perceived as rather harmful should be administered (probably after sterilization). This was pointed out by a recent study of Mai and colleagues (2007) who investigated the early microbial factors that may cause necrotizing enterocolitis in preterm infants. The latter study is unique in its experimental set-up, as samples were collected before disease, rather than collecting samples when babies are already ill. Necrotizing enterocolitis went along with a bloom in the Proteobacteria but importantly the disease was preceded by lower-than-normal levels of Proteobacteria in the early microbial population of these babies. For a proper immune maturation, both beneficial and detrimental microbes are thus necessary. Similarly, microbial network units may be designed for 3-year-old children that should be colonized by an adult-like microbiota, while also the elderly may profit from a specific microbial consortium.

Finally, microbes that may be beneficial for the entire human population may be administered to the drinking water. Recently, there were already trials to apply ‘Effective Microorganisms (EM)’ in water treatment as well as in other domains including agriculture and supplements for human consumption (Daiss *et al*., 2008; Ke *et al*., 2009). EM are mixed cultures of beneficial natural fermentative microorganisms that can be applied to increase the necessary microbial diversity needed for living. EM consist of a mixed culture of photosynthetic bacteria, lactic acid bacteria and yeast. While further research is still required to unravel the health consequences of consumption of disinfected versus undisinfected naturally colonized water, people consuming water treated with EM will presumably gain the benefit of the metabolites developed by these microorganisms, which act as substrates for increasing beneficial populations.

## Conclusions

Past and ongoing research has allowed to appreciate the importance of biodiversity in the human gut microbiome, caused by the diverse metabolic interactions among different microbial species. Rather than stimulating single species, prebiotic compounds should enhance these metabolic interactions to support a biodiverse gut microbiome. Based on the already elucidated important microbial processes within the complex human gut microbiome, future research should further aim at the elucidation and the technical formulation, production and validation of specific microbial network units.
